# BOLD-100 treatment induces systemic lipid crosstalk in metastatic colorectal carcinoma patients in a combination treatment with FOLFOX

**DOI:** 10.1016/j.isci.2026.117238

**Published:** 2026-08-24

**Authors:** Yasmin Borutzki, Gerhard Hagn, Andrea Bileck, Thomas Mohr, Mark Bazett, Malcolm Snow, Michelle Jones, Jim Pankovich, Christopher Gerner, Bernhard K. Keppler, Samuel Meier-Menches

**Affiliations:** 1 Institute of Inorganic Chemistry, Faculty of Chemistry, University of Vienna, Vienna, Austria; 2 Institute of Analytical Chemistry, Faculty of Chemistry, University of Vienna, Vienna, Austria; 3 Doctoral School in Chemistry, Faculty of Chemistry, University of Vienna, Vienna, Austria; 4 Joint Metabolome Facility, University of Vienna and Medical University of Vienna, Vienna, Austria; 5 Bold Therapeutics Inc., 170-422 Richards St., Vancouver, BC V6B 2Z4, Canada

**Keywords:** BOLD-100, Cancer, Fatty acids, FOLFOX, KP1339, Multi-omics, metals in medicine, Oxylipins, Plasma, Proteomics

## Abstract

BOLD-100 (sodium *trans*-[tetrachlorobis(1H-indazole)ruthenate(III)]) is a clinical-stage anticancer drug candidate that targets glucose-regulated protein (GRP78) and impacts the endoplasmic reticulum (ER) stress responses in cancer cells. Despite expanding knowledge about its multimodal mechanism of action from preclinical studies, little is known about the metabolic effects of BOLD-100 in patients. This study is an exploratory analysis of the blood plasma from a subset of metastatic colorectal carcinoma (mCRC) patients who were participants in a phase 1b/2a dose-escalation study (NCT04421820) using a multi-omics strategy based on proteomic and lipid analyses. BOLD-100 treatment was found to exhibit systemic, dose-dependent effects that were more pronounced on the lipid level compared with the protein level. This study indicates that BOLD-100 affects lipid profiles and influences systemic lipid crosstalk, which, in combination with oxaliplatin, leucovorin, and fluorouracil (FOLFOX) chemotherapy may, at least partially, account for the superior clinical outcomes over established therapies in mCRC patients.

## Highlights

• Exploratory biomarker study of mCRC patients treated with BOLD-100 and FOLFOX
• BOLD-100 treatment exhibits dose-dependent effects detectable in blood plasma
• BOLD-100 alters systemic lipid crosstalk in the background of FOLFOX treatment
• A prognostic biomarker signature was detected that correlates with progression-free survival

## Graphical abstract

**Figure:**
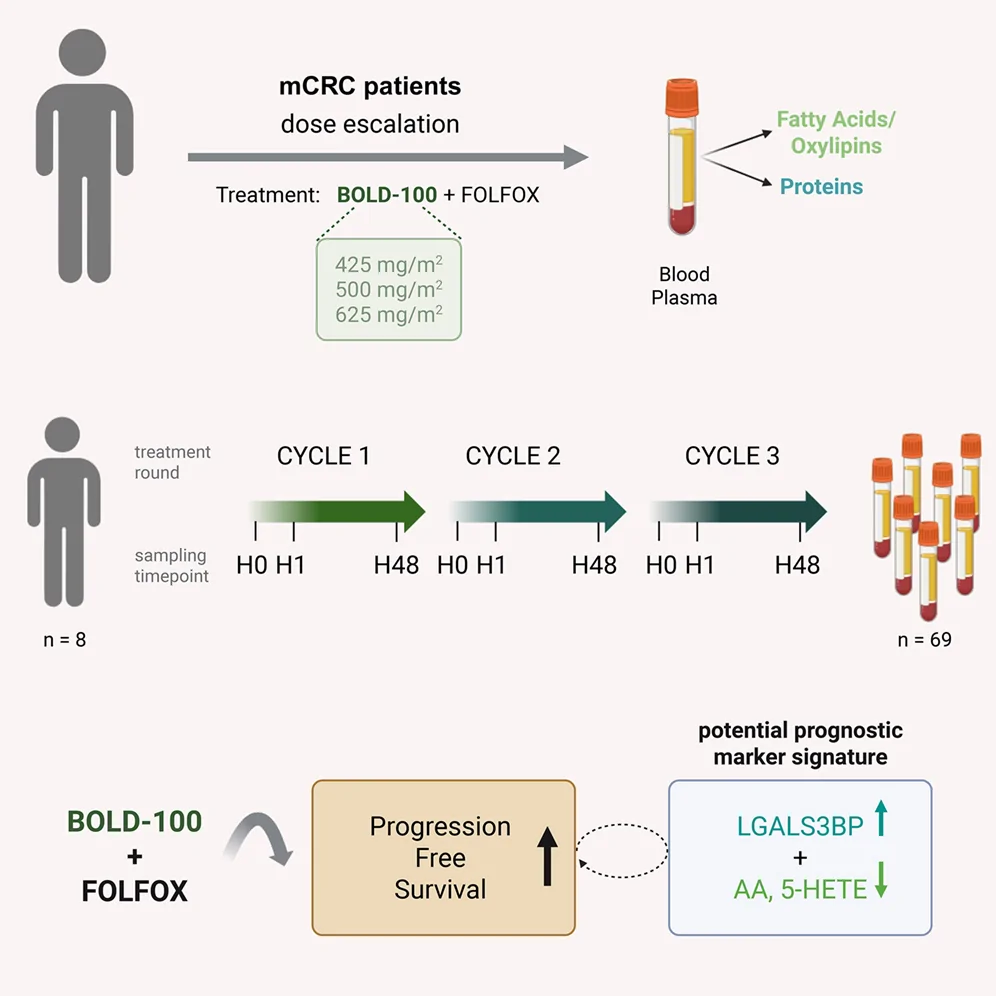

## Teaser

Cancer; Proteomics; Lipidomics

## Introduction

BOLD-100, previously known as KP-1339 or IT-139, is a clinical-stage anticancer drug candidate based on the rare metal ruthenium.^1^^,^^2^ The compound acts as an inhibitor of 78 kDa glucose-regulated protein (GRP78)^3^^,^^4^ and impacts the endoplasmic reticulum (ER) stress responses in cancer cells. A phase 1 monotherapy study was conducted, demonstrating the recommended phase 2 dose of 625 mg/m^2^, a manageable safety profile, and preliminary evidence of efficacy, particularly in patients with gastrointestinal cancers.^5^ A prospective phase 1b/2a clinical trial was then initiated to investigate the efficacy of the combination of BOLD-100 with FOLFOX chemotherapy (oxaliplatin, leucovorin, and fluorouracil) in advanced gastrointestinal cancer patients.^6^ In this dose-escalation portion of the study, the starting dose cohort was 420 mg/m^2^, and using a 3 + 3 design, was increased back to the 625 mg/m^2^ recommended phase 2 dose. The combination treatment was well tolerated and exhibited a manageable safety profile. A phase 2 expansion cohort in multiple pre-treated patients confirmed good tolerability and demonstrated in an interim analysis a significant clinical benefit over the currently available therapies against advanced metastatic colorectal carcinoma (mCRC),^7^ while promising clinical activity was reported against advanced gastric cancer^8^ and biliary tract cancer.^9^

BOLD-100 is administered intravenously and rapidly binds to plasma proteins, particularly the human serum albumin, forming a specific albumin-drug conjugate.^10^^,^^11^^,^^12^ This is thought to be responsible for the relatively long half-life of 113 h of the active ingredient observed in the blood of patients and its proposed primary hepatobiliary secretion route.^5^

Despite its selectivity for GRP78 inhibition targeting ER stress responses,^3^^,^^4^ BOLD-100 seems to follow a multimodal mechanism of action with contributions from multiple pathways. The compound induces reactive oxygen species (ROS) in cancer cells, leading to DNA damage and apoptosis.^13^^,^^14^ In colorectal cancer cells harboring an activating ^V300E^BRAF mutation, BOLD-100 specifically regulates apoptosis *via* activation of the aryl hydrocarbon receptor (AhR), ROS, and ataxia-telangiectasia and Rad3-related (ATR) signaling pathways (Ahr/ROS/ATR signaling).^15^ A chemoproteomic approach revealed that binding to ribosomal proteins accompanies ER stress induction upon BOLD-100 treatment.^16^ Independently, a sensitivity profile analysis of various cancer cell lines against BOLD-100 was performed and identified an association between ribosomal gene expression and sensitivity to BOLD-100.^17^ BOLD-100 also impacts glycolytic metabolism,^18^ possibly acting as an anti-Warburg compound, while resistance to BOLD-100 is characterized by deregulated lipid metabolism and loss of monocarboxylate transporter 1 in colon carcinoma cells.^19^ These recent findings indicate that BOLD-100 is a metabolically active drug that utilizes lipid homeostasis for its activity.

Although the multimodal mechanism of action of BOLD-100 is being elucidated in detail in preclinical models, little is known about metabolic effects in patients, especially in the context of combination treatment with FOLFOX. Therefore, employing a multi-omics strategy based on proteomic and bioactive lipid analyses, blood plasma is investigated in a subset of GI patients with advanced mCRC from the dose-escalation cohort of the registered phase 1b/2a clinical trial (NCT04421820) to explore biomarker signatures linked to systemic effects of BOLD-100 treatment. We find systemic perturbations in lipid metabolism, especially non-esterified lipids, attributable to BOLD-100 treatment and identify a potential prognostic marker signature that correlates with progression-free survival (PFS).

## Results

### Study design and cohort description

**Figure 1:**
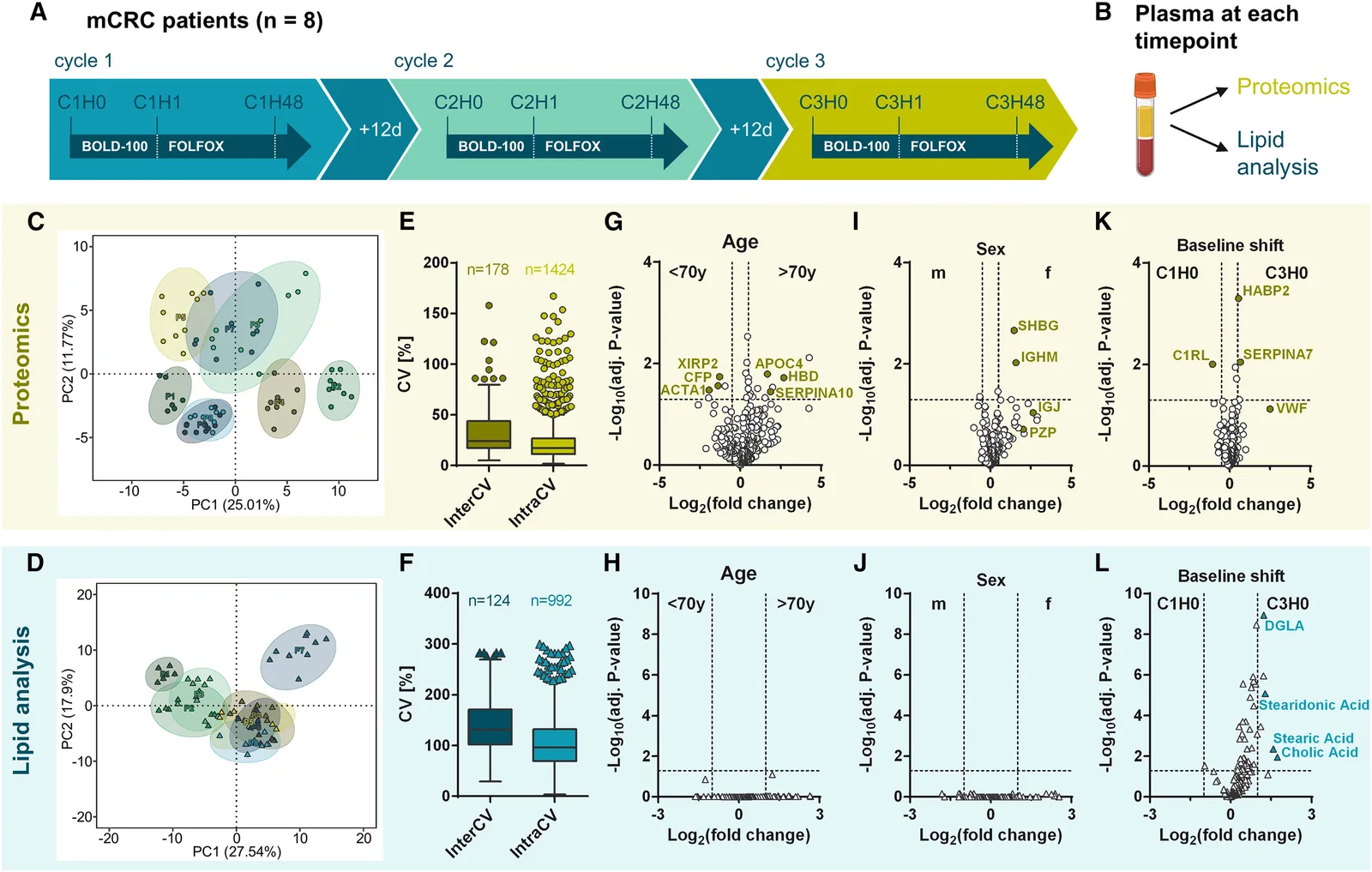
Sample collection and global characterization of plasma samples (A) Blood was collected from advanced mCRC patients (*n* = 8) during the first three cycles (C1-C3) of combined BOLD-100+FOLFOX therapy. Blood was drawn at baseline (H0), 1 h (H1), and 48 h (H48) after initiation of BOLD-100 infusion. (B) Blood plasma was obtained at each time point from C1H0–C3H48, aliquoted, and separately processed in dedicated proteomic and lipid analytical pipelines. (C) Principal component analysis of proteomic profiles of all samples (*n* = 69). Samples belonging to the same patient are shown by specific color and are grouped by an ellipsoid. (D) Principal component analysis of lipid profiles of all samples (*n* = 69). Samples belonging to the same patient are shown by specific color and are grouped by an ellipsoid. (E) Inter- and intra-patient distribution of the coefficients of variation (CV) of each detected protein. Number of data points is indicated in the figure. (F) Inter- and intra-patient distribution of the coefficients of variation (CV) of each detected lipid species. Number of data points is indicated in the figure. (G) Volcano plots to assess the potential confounding impact of age (<70 vs. >70 years, *n* = 4 per group) on the protein levels at the C3H48 time point. Statistically significant protein regulations were obtained by permutation-based multiple testing corrected *p* values, using FDR = 0.05 and log_2_ fold-change >0.5. (H) Volcano plots to assess the potential confounding impact of age (<70 vs. >70 years, *n* = 4 per group) on the lipid level at the C3H48 time point. Statistically significant regulations of lipid species were obtained by calculating *p* values of a differential expression analysis in R using the *limma* package, including multiple-testing correction by Benjamini-Hochberg, FDR = 0.05, and log_2_ fold-change >1. (I) Volcano plots to assess the potential confounding impact of sex (f vs. m, *n* = 4 per group) on the protein levelsat the C3H48 time point. Statistically significant protein regulations were obtained by permutation-based multiple testing corrected *p* values, using FDR = 0.05 and log_2_ fold-change >0.5. (J) Volcano plots to assess the potential confounding impact of sex (f vs. m, *n* = 4 per group) on the lipid level at the C3H48 time point. Statistically significant regulations of lipid species were obtained by calculating *p* values of a differential expression analysis in R using the *limma* package, including multiple-testing correction by Benjamini-Hochberg, FDR = 0.05, and log_2_ fold-change >1. (K) Volcano plots indicating baseline shifts between C1H0 and C3H0 time points (*n* = 9 per group) on the protein levels. Statistically significant protein regulations were obtained by permutation-based multiple testing corrected *p* values, using FDR = 0.05 and log_2_ fold-change >0.5. (L) Volcano plots indicating baseline shifts between C1H0 and C3H0 time points (*n* = 9 per group) on the lipid level. Statistically significant regulations of lipid species were obtained by calculating *p* values of a differential expression analysis in R using the *limma* package, including multiple-testing correction by Benjamini-Hochberg, FDR = 0.05, and log_2_ fold-change >1.

This study focuses on a subgroup of mCRC patients of a phase 1b/2a dose-escalation clinical trial of BOLD-100 in combination with FOLFOX therapy (NCT04421820). Patients 1–3 received 420 mg/m^2^, patients 4 and 5 received 500 mg/m^2^, and patients 6–8 received 625 mg/m^2^ BOLD-100 per 14-day treatment cycle. Patients received FOLFOX therapy, which was administered after completion of the BOLD-100 infusion. Patients did not have controlled diets, nor were they required to fast. Blood samples were collected over the first three cycles (C1–C3) always at baseline (H0), as well as 1 h (H1) and 48 h (H48) after initiation of BOLD-100 infusion (Figure 1A). Up to nine samples were obtained per patient. Since blood was not drawn from patient 1 at H48 in all three cycles, a total of 69 samples were obtained. Blood was collected into ethylenediaminetetraacetic acid (EDTA)-plasma tubes and processed according to a standardized protocol within 30 min. The eight mCRC patients presented with Stage IV and Eastern Cooperative Oncology Group (ECOG) performance status of 0–1. They were primarily of white ethnicity and showed an equal sex ratio (Table 1). The mean age was 62 ± 13 years for male (*n* = 4) and 69 ± 12 years for female (*n* = 4) patients (two-sided *t* test and *p* value = 0.46). Patients 1 and 6 received one, while all other patients received at least three prior systemic therapies before inclusion in this study. Patients had at least 4 treatment cycles and presented largely stable disease. A partial response was observed in patient 2, who received a total of 12 treatment cycles. The mean PFS was 5.3 months (min 1.6 and max 7.3).

**Table 1:** Clinical characteristics of the mCRC patients included in this study

N°	Sex	Age (yrs)	Ethnicity	N° prior systemic therapies	BOLD-100 dose (mg/m^2^)	N° cycles	Tumor response	Tumor mass (%)	PFS (mths)
1	m	48	White	1	420	6	SD	4	7.1
2	f	73	White	3	420	12	PR	-47	7.3
3	f	52	White	4	420	11	SD	0	6.7
4	m	66	Asian	4	500	5	SD	13	5.9
5	m	54	White	8	500	4	PD	-5	1.6
6	f	72	White	1	625	4	PD	-4	2.3
7	f	77	White	3	625	8	SD	0	5.0
8	m	78	White	6	625	9	SD	-22	6.8

The mCRC subgroup was selected from seventeen GI cancer patients of the phase 1b dose-expansion cohort. Abbreviations are as follows: m, male; f, female; SD, stable disease; PR, partial response; PD, progressive disease; PFS, progression-free survival.

### Global characterization of plasma samples

Upon thawing at 4°C, plasma samples were aliquoted and directly processed according to dedicated proteomic and lipid workflows (Figure 1B). For plasma proteomic analysis, aliquots (5 μL) were diluted, denatured and 20 μg thereof were digested in-solution. A data-dependent acquisition strategy based on label-free quantification (LFQ) proteomics was carried out. A total of 319 protein groups were identified of which 178 remained after filtering according to 75% valid values over all samples (*n* = 69). Missing values were imputed. Aliquots for bioactive lipid analysis were processed according to a solid-phase extraction (SPE) protocol using 400 μL plasma as input.^20^^,^^21^ Sample preparation included a protein precipitation step prior to enrichment so that the detected lipid species mainly correspond to the free non-esterified fraction. An untargeted data-dependent acquisition strategy was carried out on a high-resolution Orbitrap HF mass spectrometer, using an inclusion list. A total of 112 annotated lipid species were identified after manual verification and peak integration. Those correspond to the following LipidMaps^22^ classes: fatty acids + conjugates (*n* = 23, [FA01]), octadecanoids (*n* = 14, [FA02]), eicosanoids (*n* = 30, [FA03]), docosanoids (*n* = 4, [FA04]), fatty amides (*n* = 5, [FA08]), glycerophosphocholines (*n* = 16, [GP01]), glycerophosphoethanolamines (*n* = 12, [GP02]), a sphingoid base (*n* = 1, [SP01]), a steroid (*n* = 1, [ST02]), bile acids (*n* = 5, [ST04]), and a steroid conjugate (*n* = 1, [ST05]). Additionally, 32 features were included in the analysis. The list of identified lipid species can be found in Table S1. Herein, coeluting species, e.g., leukotriene B4 (LTB4)/12-epi-LTB4, were reported, as suggested recently in a position paper.^23^ Principal component analysis (PCA) of the proteomic and lipid profiles revealed that the datasets were largely determined by the individual patients (Figures 1C and 1D). The distribution of the coefficients of variation (CV) confirmed that the protein abundance is more uniform intra-patient (intra-CV and median CV = 17%) compared with inter-patient (inter-CV and median CV = 24%, Figure 1E). A similar trend was observed for the lipid species that showed higher inter-patient CV (inter-CV and median CV = 131%) compared with intra-patient CV (intra-CV and median CV = 96%, Figure 1F). Then, potential confounding effects resulting from age (<70 vs. >70 years, Figures 1G and 1H) and sex (Figures 1I and 1J) were evaluated after three treatment cycles at the C3H48 time point. No significant age and sex differences were observed on the lipid level. On the proteome level, advanced age was indicated by increased abundance of apolipoprotein C-IV (APOC4), reduced abundance of Xin actin-binding repeat-containing protein 2 (XIRP2) and alpha actin (ACTA1). Additionally, the proteome featured minor sex-specific protein differences, including sex hormone-binding globulin (SHBG), a steroid-binding protein, to be significantly elevated, while pregnancy zone protein (PZP) was elevated in female patients in some cases. B-cell-derived immunoglobulin heavy chain mu (IGHM) and immunoglobulin heavy chain J (IGJ) were also found with increased abundance in female patients. Furthermore, a negligible baseline shift was observed on the proteome level when comparing plasma protein abundances prior to therapy at C1H0 and those before the start of the third cycle at C3H0 (Figures 1K and 1L). Although not statistically significant, there was a trend of elevated levels of the von Willebrand factor (VWF) and Factor VII-activating protease (HABP2). On the lipid level, the same comparison revealed increased abundance of stearic acid (C18:0), stearidonic acid (C18:4, n-3), dihomo-γ-linolenic acid (DGLA; C20:3, n-6), and the bile acid cholic acid. The baseline shift of the lipid species manifested only after the second treatment cycle.

### Specific effects of BOLD-100 in the combination treatment

**Figure 2:**
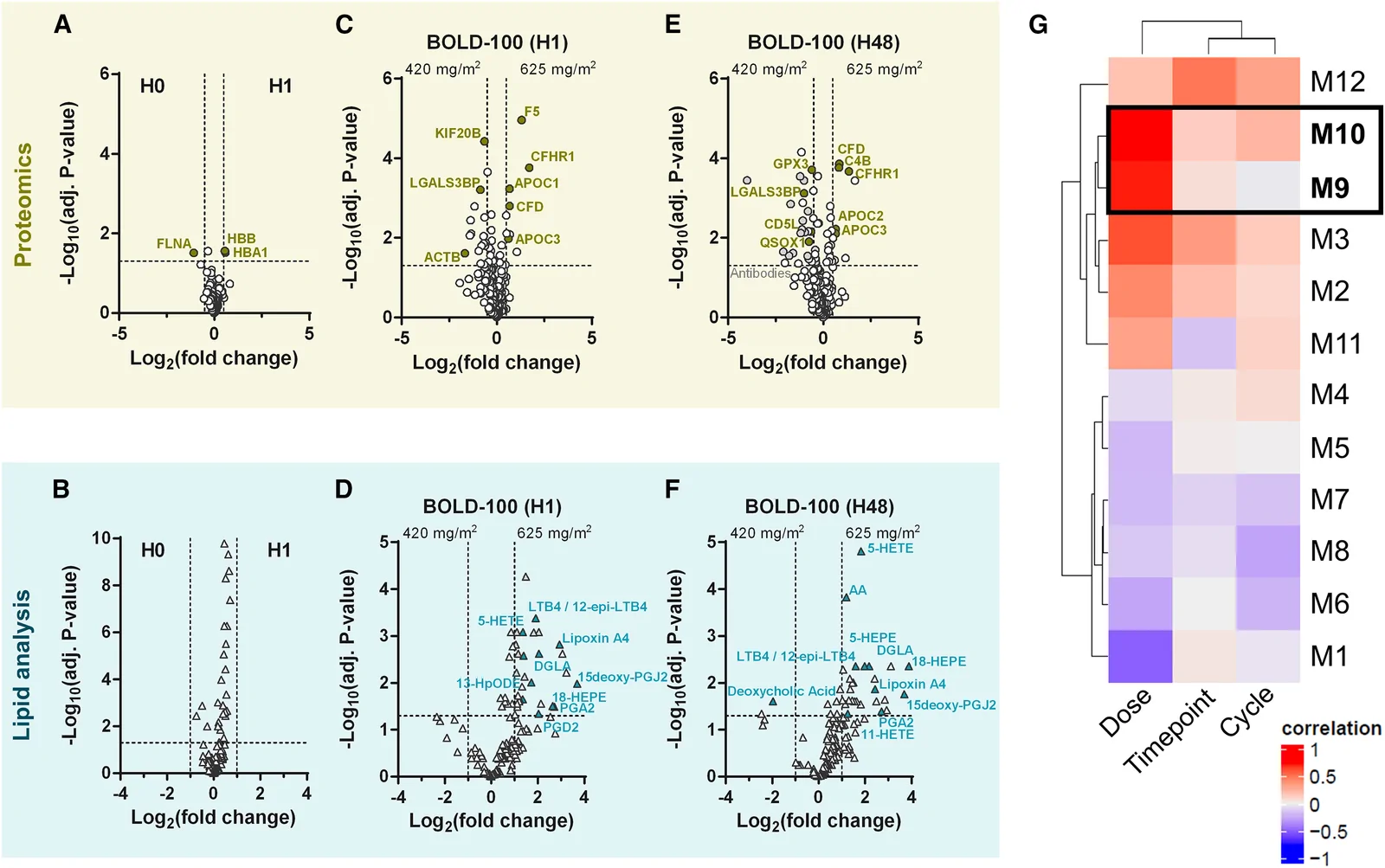
Systemic BOLD-100 effects in the combination treatment with FOLFOX in mCRC patients (A) Plasma comparison of the time point after BOLD-100 infusion, but before FOLFOX administration (H1), compared with the time point before administration (H0) across the three treatment cycles (*n* = 24 per group) on the protein level. Statistically significant protein regulations were calculated by permutation-based multiple testing corrected *p* values, using FDR = 0.05 and log_2_ fold-change >0.5. (B) Plasma comparison of the time point after BOLD-100 infusion, but before FOLFOX administration (H1), compared with the time point before administration (H0) across the three treatment cycles (*n* = 24 per group) on the lipid levels. Statistically significant regulations of lipid species were calculated from *p* values of a differential expression analysis in R using the *limma* package, including multiple-testing correction by Benjamini-Hochberg (FDR = 0.05, log_2_ fold-change >1). (C) Comparison of 625 mg/m^2^ vs. 420 mg/m^2^ BOLD-100 doses at constant FOLFOX treatment at time point H1 (*n* = 9 per group) on the level of proteins. Statistically significant protein regulations were calculated by permutation-based multiple testing corrected *p* values, using FDR = 0.05 and log_2_ fold-change >0.5. (D) Comparison of 625 mg/m^2^ vs. 420 mg/m^2^ BOLD-100 doses at constant FOLFOX treatment at time point H1 on the lipid level (*n* = 9 per group). Statistically significant regulations of lipid species were calculated from *p* values of a differential expression analysis in R using the *limma* package, including multiple-testing correction by Benjamini-Hochberg (FDR = 0.05, log_2_ fold-change >1). (E) Comparison of 625 mg/m^2^ vs. 420 mg/m^2^ BOLD-100 doses at constant FOLFOX treatment at the time point H48 on the level of proteins (*n* = 9 per group). Statistically significant protein regulations were calculated by permutation-based multiple testing corrected *p* values, using FDR = 0.05 and log_2_ fold-change >0.5. H48 samples from patient 1 were not collected (*n* = 69 samples). (F) Comparison of 625 mg/m^2^ vs. 420 mg/m^2^ BOLD-100 doses at constant FOLFOX treatment at the time point H48 on the lipid level (*n* = 9 per group). Statistically significant regulations of lipid species were calculated from *p* values of a differential expression analysis in R using the *limma* package, including multiple-testing correction by Benjamini-Hochberg (FDR = 0.05, log_2_ fold-change >1). H48 samples from patient 1 were not collected (*n* = 69 samples). (G) Co-expression analysis of the protein and lipid levels according to time point, treatment cycle, and BOLD-100 dose. Twelve modules (M1–M12) were identified that represent groups of proteins and/or lipids with coherent expression patterns (*cf*. Table S2 for detailed list). Data from the three treatment cycles were included. H48 samples from patient 1 were not collected (*n* = 69 samples).

Comparison of all H1 time points (8 patients, 3 cycles, *n* = 24) against H0 time points revealed only minor differences in the abundance of proteins or lipids (Figures 2A and 2B) that would be indicative of immediate systemic BOLD-100 effects irrespective of dose. Those included slightly elevated abundances of hemoglobin subunits alpha (HBA1) and beta (HBB). Dose-dependent effects of BOLD-100 were then analyzed by comparing patients receiving 625 mg/m^2^ (patients 6–8) with patients receiving 420 mg/m^2^ (patients 1–3) BOLD-100. Interestingly, comparing the two dose regimens revealed immediate effects at the H1 time point, some of which persisted even to the H48 time point (Figures 2C–2F). On the protein level (Figures 2C–2E), a clear signature included the elevated abundance of complement factor H-related protein 1 (CFHR1), complement factor D (CFD), and coagulation factor V (F5). Additionally, apolipoproteins C-I to C-III (APOC1–APOC3) also showed elevated abundance at the higher BOLD-100 dose. Lectin galactoside-binding soluble 3 binding protein (LGALS3BP) showed reduced abundance at the higher BOLD-100 dose from H1 to H48. Furthermore, the H48 time point revealed reduced abundances of CD5 antigen-like protein (CD5L), as well as the redox-related proteins sulfhydryl oxidase 1 (QSOX1) and glutathione peroxidase 3 (GPX3). Increased dose of BOLD-100 was characterized by increased abundances of several fatty acids and oxylipins directly after BOLD-100 administration at H1 but before FOLFOX administration (Figure 2D). Next to key markers of pro-inflammatory signaling derived from 5-lipoxygenase (5-LOX), including 5-hydroxyeicosatetraenoic acid (5-HETE), LTB4, and adrenic acid, we also observed pro-resolving markers, e.g., lipoxin A4 (LXA4), 15-deoxy-prostaglandin J2 (15d-PGJ2), 13-hydroperoxyoctadecadienoic acid (13-HpODE), 18-hydroxyeicosapentaenoic acid (18-HEPE), docosapentaenoic acid ω-3 (DPA_ω-3_), and DGLA. 13-HpODE and 18-HEPE are oxygenation products of their respective precursors, linoleic acid and eicosapentaenoic acid (EPA). Several related lysophosphatidylcholines (LPCs) and lysophosphatidylethanolamines (LPEs) were also upregulated, including LPC (0:0/22:4), LPC (0:0/22:5), LPC (0:0/22:6), LPE (0:0/20:4), LPE (0:0/22:6), and LPE (22:6/0:0). Some molecules of this signature persisted to the H48 time point (Figure 2F), especially 5-HETE and LTB4, but also LXA4 and 18-HEPE. After completed administration of BOLD-100+FOLFOX at H48, arachidonic acid (AA), 11-hydroxyeicosatetraenoic acid (11-HETE), and 5-hydroxyeicosapentaenoic acid (5-HEPE) were also upregulated. Of the LPCs and LPEs, we found LPC (0:0/22:6) and LPE (22:6/0:0) still upregulated at H48. Finally, the secondary bile acid deoxycholic acid and docosahexaenoic acid (DHA) were downregulated at this time point. A weighted co-expression network analysis of the proteomic and lipid data was then performed. Twelve modules (M1–M12) were identified that represent groups of proteins and/or lipid species with coherent regulation patterns and those were investigated with respect to their correlation to treatment cycle, blood collection time points, and BOLD-100 doses (Figure 2G). The detailed list of proteins and lipid species per module can be found in Table S2. Interestingly, BOLD-100 dose showed stronger correlation patterns compared with time point or cycle. Especially, modules 10, 9, and 3 exhibited substantial positive correlation. Module 10 contained exclusively fatty acids and oxylipins, including 13-HpODE and 5-HETE. Module 9 included APOC1 and the pro-resolving mediators 15d-PGJ2, LXA4, and 18-HEPE. Module 3 showed a slightly lower correlation factor and included some LPCs and LPEs, as well as c-reactive protein (CRP). Therefore, BOLD-100 dose seemed to have a stronger impact on lipid species than proteins. Module 1 demonstrated the strongest anticorrelation with BOLD-100 dose. Components of this module mainly included antibodies and plasma proteins related to extracellular exosome formation (Table S2).

### Prognostic marker signature for progression-free survival

**Figure 3:**
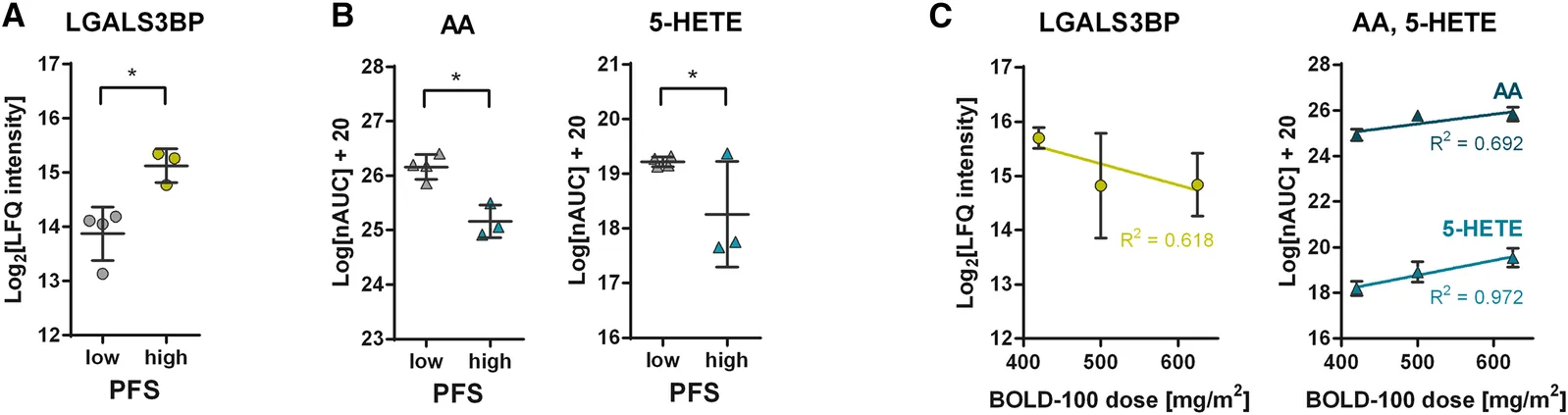
Potential prognostic marker signature of BOLD-100 treatment (A) Potential prognostic protein biomarker at the last collection time point (C3H48) that correlates with progression-free survival (PFS). High PFS, patients 2, 3, and 8; low PFS, patients 4–7. Patient 1 sample (high PFS) was not collected at C3H48. ∗*p* value < 0.05. Statistically significant protein regulation was calculated by permutation-based multiple testing corrected *p* values, using FDR = 0.05 and log_2_ fold-change >0.5. Data are represented as mean ± standard deviation. Individual data points are shown. (B) Potential prognostic lipid biomarkers at the last collection time point (C3H48) that correlate with progression-free survival (PFS). High PFS, patients 2, 3, and 8; low PFS, Patients 4–7. Patient 1 sample (high PFS) was not collected at C3H48. ∗*p* value < 0.05. Statistically significant regulations of lipid species were calculated from a differential expression analysis in R using the *limma* package, including multiple-testing correction by Benjamini-Hochberg (FDR = 0.05, log_2_ fold-change >1). Data are represented as mean ± standard deviation. Individual data points are shown. (C) The potential biomarkers show a dose-dependent abundance pattern at the H1 time point after BOLD-100 treatment but before FOLFOX administration. The coefficient of determination is provided (R^2^). *n*(420 mg/m^2^ dose) = 9; *n*(500 mg/m^2^ dose) = 6; *n*(625 mg/m^2^ dose) = 9. Data are represented as mean ± standard deviation.

The BOLD-100+FOLFOX combination treatment was shown to improve PFS in mCRC patients compared with the existing therapy.^7^ To evaluate prognostic markers for improved PFS, the patients were separated into PFS low (1.6–5.9 months, *n* = 4) and high groups (6.7–7.3 months, *n* = 3). Patient 1 (PFS = 7.1 months) was excluded from this analysis because H48 time points were not available. Marker analysis was carried out at the last time point C3H48 after three complete treatment cycles. Importantly, potential prognostic marker molecules were identified on both protein and lipid levels, including LGALS3BP, AA, and 5-HETE. PFS was positively associated with LGALS3BP abundance, while it was negatively associated with AA and 5-HETE (Figures 3A and 3B). These three potential biomarkers show an inverse dose-dependent abundance pattern compared with PFS (Figure 3C).

## Discussion

A phase 1b/2a dose-escalation study was initiated with BOLD-100 in combination with FOLFOX in patients with advanced gastrointestinal (GI) cancer.^6^ The combination treatment is well tolerated and shows a manageable safety profile. Additional phase 2 expansion cohorts with advanced mCRC,^7^ gastric cancer^8^ and biliary tract cancer^9^ patients showed superiority over currently available therapies, especially in the case of mCRC. This is noteworthy, since some of the patients had already undergone prior treatment with FOLFOX. To increase statistical power due to the small number of mCRC patients included in the dose-escalation cohort, blood samples were collected over the first three cycles. At each cycle, plasma was collected before administration (H0), as well as 1 h (H1) and 48 h (H48) after initiation of the BOLD-100 infusion (Figure 1A). This increases the data points for each time point from *n* = 8 to *n* = 24. Blood plasma was preferred over serum for biomarker studies due to the absence of blood clotting and therefore negligible confounding impact of potential co-administered anti-thrombotic agents, which was not controlled for.^20^ Each plasma sample was aliquoted and processed with dedicated proteomic and lipid workflows (Figure 1B).^20^^,^^24^ Lipid species were obtained from the non-esterified fraction of the blood plasma after protein precipitation.

The proteome and lipid profiles were largely determined by the individual patients, indicating pronounced inter-individual variability (Figures 1C and 1D). This was also reflected in higher inter-patient variation compared to intra-patient variation (Figures 1E and 1F). Since biomolecule CVs were calculated across all time points, the increased CV distribution of the lipid species indicated a more pronounced variation in the frame of the treatment. There was negligible impact of age (Figures 1G and 1H) or sex (Figures 1I and 1J) on protein and lipid levels in this cohort. On the proteome level, age increased the abundance of APOC4, a minor apolipoprotein that was already associated with age.^25^ Reduced abundance of ACTA1 and the actin-capping XIRP2 may indicate lower muscle turnover with advanced age. SHBG and PZP are known sex-specific proteins, and the former was consistently found at elevated abundance in female patients.^26^ There was also a negligible baseline shift between BOLD-100+FOLFOX treatment-naive patients and after two cycles of therapy (Figures 1K and 1L). Although not significant, the acute-phase inflammation marker VWF was slightly elevated after two treatment cycles, similarly to HABP2, which was identified as a tumor suppressor in thyroid cancer.^27^ These evaluations informed about potential confounding factors, which were accounted for in further data analysis. The plasma protein and lipid levels after BOLD-100 infusion (H1) revealed no statistically significant regulation of lipid species compared to baseline across all patients, but slightly increased hemoglobin subunits HBB and HBA1 (Figures 2A and 2B), indicating a minor hemolytic effect on erythrocytes.^28^ This is coherent with the slightly elevated VWF (see aforementioned).

Characterizing drug effects of specific components in a combination treatment poses a significant challenge, especially in the context of clinical studies. In this study, for example, molecular profiles of samples from the H48 time point contain potential contributions from four active agents, including BOLD-100 and the three FOLFOX agents. Notably, the treatment schedule involved infusion of BOLD-100 prior to FOLFOX administration. Therefore, the H1 time point in plasma indicates immediate BOLD-100-specific effects. However, comparing all H1 samples with H0 samples did not reveal major differences of BOLD-100 treatment irrespective of dose (Figures 2A and 2B). Since this phase 1b/2a included a 3 + 3 dose escalation design with GI cancer patients, mCRC patients were treated with 420 mg/m^2^ (P1–P3) or 625 mg/m^2^ (P6–P8) BOLD-100. Because FOLFOX doses remained constant for all patients, directly comparing the two BOLD-100 doses enables the elucidation of dose-dependent BOLD-100 effects largely cleared of the systemic impact of FOLFOX therapy. Indeed, using this comparison, a considerable number of BOLD-100 effects were observed on both protein (Figures 2C and E) and lipid levels (Figures 2D and F) at the H1 and H48 time points. Additionally, BOLD-100 dose also had the strongest impact in the co-correlation analysis (Figure 2G). BOLD-100 seems to affect the lipid level more strongly compared with the protein level.

In detail, dose-dependent effects on the protein level were observed at H1 and H48 (Figures 2C and 2E) and included increased abundance of CFD, CFHR1, and APOC1–APOC3, while LGALS3BP and CD5L showed reduced abundance. CFD is a component of the alternative activation pathway of the complement system and is mainly produced by adipocytes, controlling immune responses and systemic energy metabolism, including lipolysis in adipocytes, triglyceride synthesis, and glucose uptake.^29^^,^^30^ CFHR1 competes with complement factor H (CFH), an inhibitor of the complement alternative pathway,^31^ and can associate with lipoproteins, especially low-density lipoprotein (LDL).^32^ At H1 (Figure 2C), upregulated APOC1 is known to bind circulating free fatty acids and appears to interfere with fatty acid uptake.^33^^,^^34^ It further inhibits very-low-density lipoproteins (VLDL) from binding to (V)LDL receptors.^34^^,^^35^ CD5L (also known as AIM) is a critical immune and lipid metabolism regulator.^36^^,^^37^ It is also known to protect macrophages from oxidized lipids.^38^ At H48 (Figure 2E), upregulated APOC2 may act as an activator of lipoprotein lipase to release free fatty acids in blood.^39^ At the same time point, upregulated APOC3 inhibits lipoprotein lipase.^40^ Both APOC2 and APOC3 are associated with triglyceride-rich lipoproteins, such as VLDLs.^41^ Interestingly, LGALS3BP (formerly known as Mac-2-BP) was previously identified to bind to tumor-secreted antigens and has immunostimulatory activity,^42^ especially *via* pattern-recognition receptor-mediated signal transduction.^43^ Thus, the protein signature of BOLD-100 treatment seems to indicate an influence on the adipocyte-lipoprotein-immune axis to regulate free fatty acids.

Interestingly, the non-esterified fraction of the bioactive lipids revealed a minor baseline shift over the three therapy cycles toward increased fatty acids, including DGLA, stearidonic acid, and stearic acid (Figure 1L). The dose-dependent lipid profiles featured numerous upregulated lipid species irrespective of the time point (Figures 2D and 2F). Classical AA-derived oxylipins were upregulated at H1, including the 5-lipoxygenase (5-LOX) products 5-HETE and LTB4.^44^ These two eicosanoids are well-known neutrophil-derived chemoattractants. Prostaglandin D2 (PGD2) is an AA product that is mainly produced by macrophages *via* cyclooxygenase-2 (COX-2) activity. Next to these lipid species, we also found 5-LOX-derived 5-HEPE and LXA4 to be upregulated. The former is an oxygenation product of EPA and modulates neutrophil response, while the latter represents a pro-resolving mediator that promotes macrophage phagocytosis.^45^ Similarly, 15d-PGJ2 is a bioactive lipid mediator and terminal degradation product of PGD2. Being an endogenous ligand of peroxisome proliferator-activated receptor-γ (PPAR-γ) receptor, it can modulate inflammatory responses and regulate the expression of proteins related to lipid metabolism.^46^ Importantly, 15d-PGJ2 was previously shown to exert anticancer activity in colon cancer cells *via* PPAR-γ activation and upregulation of *c-jun*.^47^ Consequently, BOLD-100 treatment seemed to induce a mixed response of lipid species, such as fatty acids, oxylipins, bile acids and lysolipids, in mCRC patients that is driven by the simultaneous upregulation of acute-phase inflammatory and pro-resolving mediators.^44^ While the inflammatory response may be triggered by hemolysis of erythrocytes (Figure 2A) or other damage-associated molecular patterns, the origin of the pro-resolving mediators remains speculative. However, this response is clearly different from a lipopolysaccharide-stimulated inflammatory response.^21^ The PPAR-γ ligand 15d-PGJ2 may represent a potential candidate to explain the lipid-mediated antitumor effects of BOLD-100 treatment specifically.

Next, potential prognostic markers were evaluated that are indicative of clinical benefit of the combination therapy, especially PFS. LGALS3BP, AA, and 5-HETE showed significant differences between high- and low-PFS groups (Figures 3A and 3B). The three molecules were all found to correlate specifically with BOLD-100 treatment, featuring R^2^ > 0.62 (Figure 3C). While AA and 5-HETE abundances correlated positively and LGALS3BP negatively with BOLD-100 dose, this trend was inverted with respect to the prognostic signature for high PFS. As described earlier, increased LGALS3BP may be associated with immunostimulatory activity,^42^ and can activate nuclear factor kappa-light-chain-enhancer of B cells (NF-κB) pathways,^43^ while down-regulated AA and 5-HETE may explain a detrimental effect of pro-inflammatory factors on PFS. Consequently, elevated LGALS3BP with concomitantly reduced AA and 5-HETE levels may serve as a prognostic signature correlating with PFS in the combination treatment of mCRC patients with BOLD-100+FOLFOX.

In summary, employing a plasma multi-omics strategy based on proteomic and lipid analyses, BOLD-100 was found to induce a more pronounced dose-dependent effect on the lipid level compared to the protein level in this mCRC subgroup. Co-expression analysis revealed a pronounced impact of BOLD-100 dose on plasma profiles, which seem to converge on the systemic regulation of lipid metabolism. This regulation is complex and indicates altered crosstalk between adipocytes, liver-derived lipoproteins and immune cells. Furthermore, increased abundance of LGALS3BP and decreased abundance of AA and 5-HETE may represent a prognostic marker signature for PFS, which will be verified in a larger prospective cohort. Therefore, the clinical benefit of BOLD-100 in combination with FOLFOX therapy seems to be partially explained by its impact on systemic crosstalk of lipid metabolism.

### Limitations of the study

This clinical trial investigates BOLD-100 in combination with the chemotherapy regimen FOLFOX. The trial was designed with an initial dose escalation in a multi-arm 3 + 3 design, followed by a dose-expansion cohort. The multi-arm design included several GI cancer types, including gastric cancer, pancreatic cancer, cholangiocarcinoma, and mCRC. The mCRC patients were the largest subgroup, but the relatively small number of those patients in the dose-escalation part represents a limitation for this exploratory study. Consequently, we opted to collect blood samples over 3 therapy cycles for each patient. Therefore, each patient sampling time point is represented by three data points from cycles 1–3, which increases statistical power. Furthermore, metabolic parameters, including lipid species, are influenced by diet. As the trial was primarily focused on safety and efficacy endpoints in advanced cancer patients, diet was not controlled. Subsequent investigations with controlled diets would be required to confirm these results. This trial did not control for other illnesses and concomitant therapies that might impact metabolic factors. As an octahedral ruthenium(III) complex with coordinated chloride and indazole ligands,^1^ BOLD-100 undergoes ligand-exchange-mediated metabolism, instead of classic phase I/II biotransformation, and future studies will be required to characterize its impact on hepatic cytochrome P450 function. Finally, while renal clearance is minimal for BOLD-100,^5^ evidence about other elimination pathways also requires further investigations.

## Resource availability

### Lead contact

Further information and requests for resources should be directed to and will be fulfilled by the lead contact, Samuel Meier-Menches (samuel.meier-menches@univie.ac.at).

### Materials availability

This study did not generate new unique reagents.

### Data and code availability

• The lipid data were submitted to the MassIVE data repository, which is a full member of the ProteomXchange consortium, and can be accessed under dataset identifier MassIVE: MSV000099389 (https://doi.org/10.25345/C5WS8J043). Proteomic data were submitted to the PRIDE partner repository, also a full member of the ProteomXchange consortium, and can be accessed under dataset identifier: PXD069599. Data associated with this study are provided in the main text or Table S1. Any additional information required to reanalyze the data reported in this paper is available from the lead contact upon request.
• This paper does not report original code.

## Acknowledgments

The authors are grateful to the Core Facility of Mass Spectrometry at the Faculty of Chemistry (University of Vienna) and the Joint Metabolome Facility (Medical University of Vienna and University of Vienna). Both are members of the Vienna Life-Science Instruments (VLSI) initiative.

## Author contributions

Conceptualization, M.B., J.P., C.G., B.K.K., and S.M.-M.; methodology, M.B., M.J., J.P., and S.M.-M.; investigation, Y.B., G.H., A.B., and T.M.; visualization, Y.B., T.M., and S.M.-M.; project administration, M.B., J.P., B.K.K., and S.M.-M.; supervision, M.B., B.K.K., S.M.-M., and C.G.; writing – original draft, Y.B., and S.M.-M.; writing – review and editing, all authors.

## Declaration of interests

J.P. is co-founder of Bold Therapeutics Inc. J.P., M.B., M.S., and M.J. are employees of Bold Therapeutics Inc.

## Declaration of generative AI and AI-assisted technologies in the writing process

During the preparation of this work, the authors used DeepL.com to improve English style. After using this tool or service, the authors reviewed and edited the content as needed and take full responsibility for the content of the publication.

## STAR★Methods

### Key resources table

**Table:** 

REAGENT or RESOURCE	SOURCE	IDENTIFIER
**Biological samples**		

Blood plasma from mCRC patients participating in a clinical phase 1b/2a study	This paper	Clinical trial https://clinicaltrials.gov/study/NCT04421820

**Chemicals, peptides, and recombinant proteins**		

Plasma preparation tube with K_2_EDTA anticoagulant	BD Bioscience	Cat# 362799
Cryotubes, 2 mL	Greiner Bio-One	Cat# 121280
Sodium deoxycholate buffer, SDC	GERBU Biotechnik GmbH	Cat# D520
Water (H_2_O) LC-MS CHROMASOLV™	Honeywell Riedel-de-Haën	Cat# 15665350
Water (only used for SPE washing)	VWR Chemicals	Cat#83645.320
Tris·HCl (pH 8.3)	GERBU Biotechnik GmbH	Cat# 1480
Bicinchoninic acid (BCA)	Sigma-Aldrich	Cat# D8284
Tris(2-carboxyethyl)phosphine (TCEP)	Sigma-Aldrich	Cat# C4706
2-chloroacetamide (2-CAM)	Alfa Aesar	Cat# 11462278
Trypsin/Lys-C Mix, Mass Spec Grade	Promega GmbH	Cat# V5071
Isopropanol	VWR Chemicals	Cat# 20839
Trifluoroacetic acid (TFA)	Sigma Aldrich	Cat# 302031
Empore™ Styrene divinylbenzene-reverse phase sulfonate (SDB-RPS)	CDS Analytical	Cat# 98-0604-0226-4 EA
Acetonitrile (ACN) LC-MS CHROMASOLV™, ≥99.9%	Honeywell Riedel-de-Haën	Cat# 34967
NH_4_OH	Sigma-Aldrich	Cat# 74292
Formic acid (LC-MS grade)	VWR Chemicals	Cat# 84865.180
Ethanol (EtOH) (HPLC grade)	VWR Chemicals	Cat# 20825.324
StrataX solid-phase extraction (SPE) columns (30 mg mL^−1^)	Phenomenex	Cat# 8B-S100-TAK
Methanol (MeOH), MS grade	VWR Chemicals	Cat# 34966

**Deposited data**		

Proteomics Data	This paper	PRIDE PXD069599
Bioactive Lipid Data	This paper	MassIVE: MSV000099389

**Software and algorithms**		

Xcalibur Qual Browser (version 4.1.31.9)	Thermo Fisher Scientific	thermo fischer userguide:https://documents.thermofisher.com/TFS-Assets/CMD/Package-Inserts/man-xcali-97929-xcalibur-41-xreport-manxcali97929-en.pdf
LIPIDMAPS repository, July 2020	N/A	Cockayne et al.^48^
TraceFinder software (version 4.1)	Thermo Fisher Scientific	https://www.thermofisher.com/at/en/home/industrial/mass-spectrometry/liquid-chromatography-mass-spectrometry-lc-ms/lc-ms-software/lc-ms-data-acquisition-software/tracefinder-software.html
R (version 4.2.0)	http://www.rstudio.com/	
imputeLCMD package (version 2.1)	in R (version 4.2.0)	Lazar et al.^49^
Limma package (version 3.50.0)	in R (version 4.2.0)	Ritchie et al.^50^
Ggplot2 package (version 3.4.0)	in R (version 4.2.0)	Wickham et al.^51^
WGCNA (version 1.74)	in R (version 4.2.0)	Langfelder et al.^52^

### Experimental model and study participant details

BOLD-100-001 (Code: NCT04421820) is an open-label Phase 1b/2a dose-escalation trial of BOLD-100 in combination with FOLFOX therapy for the treatment of patients with advanced gastrointestinal cancers who have failed at least one prior line of systemic therapy in the advanced setting.^6^ The Phase 1b portion of the study was designed as a multi-arm open label 3 + 3 dose expansion focusing on GI cancers, including gastric cancer (*n* = 1), pancreatic cancer (*n* = 4), cholangiocarcinoma (*n* = 4) and mCRC patients (*n* = 8). Seventeen patients were included in this phase 1b portion and plasma samples were obtained from the subset of eight mCRC patients for this exploratory biomarker study. Eligibility criteria are detailed in full at clinicaltrials.gov/study/NCT04421820. In brief, inclusion criteria were as follows: 18 years or older, presenting histologically/cytologically confirmed GI tumors that are metastatic or unresectable, be ambulatory with ECOG performance score of 0/1, be on stable doses of any drugs that may affect hepatic drug metabolism or renal drug excretion. Exclusion criteria were neuropathy grade >2, any other known malignancy within 3 years before start of treatment, active GI tract disease with malabsorption syndrome or non-healing wounds, among others. All participants provided written informed consent prior to inclusion in accordance with the Declaration of Helsinki. Ethical approval was granted by local Institutional Review Boards and Research Ethics Boards at each participating clinical site, including the Cross Cancer Institute (Health Research Ethics Board of Alberta, Ethics ID: HREBA.CC-20-0157), Juravinski Cancer Center (Ontario Cancer Research Ethics Board, CTO Project ID: 2113), and Jewish General Hospital (CIUSSS West-Central Montreal, Project ID: MEO-37-2021-2421). An independent data monitoring committee reviewed safety data. The trial was funded by Bold Therapeutics Inc. Patients received BOLD-100 doses of 420 (patients 1 to 3), 500 (patients 4, 5) or 625 mg/m^2^ (patients 6 to 8) per treatment cycle. BOLD-100 was administered by intravenous infusion for 60–90 min before administering FOLFOX chemotherapy. For the FOLFOX regimen, oxaliplatin (85 mg/m^2^) and leucovorin (400 mg/m^2^) were administered for 2 h before a bolus fluorouracil (400 mg/m^2^). Subsequently, fluorouracil (2400 mg/m^2^) was continuously infused over 46 h. The treatment cycles were every two weeks. Blood samples were collected over the first three treatment cycles (C1–3). In each cycle, samples were collected before administration (H0), and 1 h (H1) or 48 h (H48) after initiation of the BOLD-100 infusion. Up to nine samples were collected from each patient and a total of 69 samples were obtained. BOLD-100 infusion typically started between 8 and 11 am. The clinical characteristics of the patients are shown in Table 1. There were equal numbers of both sexes and sex had a minor influence on the proteome profile.

### Method details

#### Plasma collection

Blood was collected from patients by drawing blood into plasma preparation tubes (PPT, 5 mL, K_2_EDTA anticoagulant, BD Biosciences). The tubes were gently inverted 8–10 times and samples were centrifuged within 30 min (1100 g, 10 min, room temperature (RT)). The plasma was aliquoted on ice into cryotubes (1 mL) and was immediately frozen in a −80°C freezer, where they were stored. Samples were then thawed at +4°C, aliquoted and directly processed in dedicated proteomic (5 μL aliquot) and lipid (400 μL aliquot) workflows.

#### Proteomics

Plasma aliquots (5 μL) were diluted 1:20 with sodium deoxycholate buffer (SDC; 0.4 g SDC, 500 μL Tris·HCl (pH 8.8) in 9.5 mL H_2_O) and heated to 95°C in a Thermo Shaker (Eppendorf, 5 min, 1400 rpm). After cooling to RT, the protein amount was quantified using a conventional bicinchoninic acid assay. An aliquot corresponding to 20 μg protein of each sample was transferred in an Eppendorf tube and digested in-solution.^53^ In a first step, the proteins were reduced and alkylated with 10 μL of a mixture containing 100 mM tris(2-carboxyethyl)phosphine (TCEP) and 400 mM 2-chloroacetamide (2-CAM) for 5 min at 45°C, followed by 18 h digestion with Trypsin/Lys-C (1:100 enzyme-to-substrate ratio) at 37°C. The samples were reconstituted in styrene divinylbenzene-reverse phase sulfonate (SDB-RPS) loading buffer consisting of 99% isopropanol and 1% trifluoroacetic acid (TFA) and then desalted using SDB-RPS StageTips. Peptides were eluted with freshly prepared SDB-RPS elution buffer (2.4 mL acetonitrile (ACN), 1.6 mL H_2_O, 20 μL NH_4_OH), completely dried in a SpeedVac. Peptide samples were reconstituted in 5 μL formic acid (30%) containing synthetic standard peptides and subsequently diluted with 40 μL of loading solvent (97.95% H_2_O, 2% ACN and 0.05% TFA).

Nanoflow liquid chromatography-tandem mass spectrometry (nLC-MS/MS) was carried out on a timsTOF Pro mass spectrometer (Bruker Daltonics, Bremen, Germany) hyphenated to a Dionex UltiMate^TM^ 3000 RSLCnano system (Thermo Scientific, Bremen, Germany). Samples (1 μL injection volume) were loaded on an Acclaim^TM^PepMap^TM^ C18 HPLC pre-column (2 cm × 100 μm, 100 Å, Thermo Fisher Scientific, Vienna, Austria). An Aurora series CSI UHPLC emitter column (25 cm × 75 μm, 1.6 μm C18, IonOpticks, Fitzroy, Australia) was used for chromatographic separation and a gradient from 7 to 40% of mobile phase B (79.9% ACN, 20% H_2_O, 0.1% formic acid (FA)) and mobile phase A (99.9% H_2_O, 0.1% FA) was applied. The flow rate for trapping was 10 μL min^−1^, the flow rate for chromatographic separation was 300 nL min^−1^. Samples were analyzed using data-dependent acquisition and LFQ shotgun proteomics in PASEF mode.^20^ Plasma samples were analyzed using a 43 min gradient with a total run time of 85 min^20^

#### Lipid analysis

Plasma (400 μL) was diluted 1 : 5 with ice-cold ethanol (EtOH, HPLC grade, VWR, −20°C) in Falcon tubes (15 mL volume) to precipitate proteins and stored at −20°C overnight. For processing, the samples were centrifuged (30 min, 4536 g, 4°C) and the clear supernatants were transferred into fresh Falcon tubes. EtOH was evaporated in a SpeedVac until the original sample volume was restored. Samples were loaded onto preconditioned StrataX SPE columns (30 mg mL^−1^, Phenomenex) using Pasteur pipettes. Columns were washed with cold water (5 mL, MS grade, VWR) and analytes were eluted with cold methanol (MeOH, 500 μL, MS grade, VWR) containing 2% formic acid. The samples were then dried under nitrogen at RT and reconstituted in 150 μL reconstitution buffer (H_2_O: ACN: MeOH [vol % 65 : 31.5: 3.5] + 0.2% FA). This procedure allows the analysis of lipids, such as non-esterified fatty acids, oxylipins, bile acids, steroids, lysolipids, endocannabinoids, and related molecules, in blood plasma (cf. Lipid raw data processing for annotated lipid species).

Liquid chromatography-tandem mass spectrometry (LC-MS/MS) was carried out on a high-resolution quadrupole orbitrap mass spectrometer (Thermo Fisher Scientific QExactive HF), equipped with a HESI source. The instrument was operated in negative ionization mode for oxylipins, fatty acids and conjugates, bile acids, lysolipids and sphingosine-1-phosphate, and in positive mode for cortisol, endocannabinoids and related molecules. The MS parameters were as follows: Spray voltage 3.5 kV, capillary temperature 253°C, sheath gas 46 a.u. and auxiliary gas 10 a.u., mass range *m/z* 250–700 on the MS1 level (60′000 resolution at *m/z* 200). A Top2 method with HCD fragmentation and normalized collision energy of 24 was used for data-dependent acquisition. MS/MS resolution was 15′000. Data was acquired based on an inclusion list with 33 precursor masses, which are specific for oxylipins and their precursor fatty acids.^53^ Chromatographic separation was achieved on a Vanquish UHPLC system (Thermo Fisher Scientific) equipped with a reversed-phase Kinetex® XB-C18 column (2.6 μm XB-C18, 100 Å, LC column 150 × 2.1 mm, Phenomenex). Samples (20 μL injection volume) were acquired in technical duplicates in negative ion mode, and once in positive ion mode. Flow rate for chromatographic separation was 200 μL min^−1^. The LC column oven was set to 40°C and the autosampler was set to 4°C. Eluents were H_2_O + 0.2% FA (Eluent A), mobile phase B: ACN: MeOH [vol % 90 : 10] + 0.2% FA (Eluent B). A gradient flow was used from 35 to 90% B (1–10 min). The total run time of 20 min included washing and equilibration.

### Quantification and statistical analysis

#### Proteomic raw data processing

Raw data obtained from the timsTOF Pro MS was processed based on LFQ proteomics by MaxQuant (Version 1.6.17.0),^54^ including the built-in Andromeda search engine, and searched against the UniProt Database.^55^ The search was performed using a fasta file for the human proteome (version 11/2021, 20′375 entries). Only non-redundant Swissprot entries with at least two identified peptides per protein were used for identifying protein groups. The first and main search peptide tolerance was 50 and 25 ppm, respectively. The false discovery rate (FDR) was fixed to 0.01 on the peptide and protein level. Match between runs was enabled with an alignment time window of 0.7 min. Oxidation of methionine and N-terminal acetylation were set as variable modifications whereas carbamidomethylation of cysteines was set as fixed modification. The statistical evaluation was performed with Perseus (Version 1.6.14) using LFQ intensities of the MaxQuant result file. After filtering potential contaminants, the LFQ values were log_2_-transformed. Only those protein groups with ≥75% valid values in total were considered for data evaluation. Missing data points were imputed by a standard approach using a left-censored Gaussian distribution (width 0.3, down-shift 1.8).

#### Lipid raw data processing

Raw files were checked using the Xcalibur Qual Browser (version 4.1.31.9, Thermo Fisher Scientific) by comparison to reference spectra (LIPIDMAPS repository,^22^ the LIPIDMAPS standard spectral library^48^ and in-house measured standards). Analytes were identified based on exact mass, retention time (±0.05 min) and MS/MS fragmentation pattern with at least 3 matching product ions. The TraceFinder software (version 4.1, Thermo Fisher Scientific) was applied for relative quantification, allowing a mass deviation of 5 ppm. A total of 112 annotated lipid species (fatty acids + conjugates (*n* = 23, [FA01]), octadecanoids (*n* = 14, [FA02]), eicosanoids (*n* = 30, [FA03]), docosanoids (*n* = 4, [FA04]), fatty amides (*n* = 5, [FA08]), glycerophosphocholines (*n* = 16, [GP01]), glyerophosphoethanolamines (*n* = 12, [GP02]), a sphingoid base (*n* = 1, [SP01]), a steroid (*n* = 1, [ST02]), bile acids (*n* = 5, [ST04]) and a steroid conjugate (*n* = 1, [ST05])) and 32 features were included in the analysis. The resulting data containing peak areas of each analyte were loaded into the R software package environment (version 4.2.0) and were log_2_-transformed. Then, the log_2_-transformed peak area of the internal standards was subtracted from the log_2_-transformed analyte peak areas and further transformed (x+20) to obtain a similar value distribution compared to LFQ proteomics. Missing values were imputed using the minProb function of the imputeLCMD package (version 2.1).^49^ Differential expression analysis was performed using the *limma* package (version 3.50.0)^50^ with multiple grouping variables (blood sampling timepoint, patient’s sex, BOLD-100 dose, patient’s age), including a blocking factor for paired measurements. Intrablock correlation was estimated with “duplicateCorrelation”, linear models were fitted and moderated using “lmFit” and “eBayes”. Volcano plots were produced using the ggplot2 package (version 3.4.0).^51^

#### Weighted co-expression network analysis (WGCNA)

After separate data preprocessing of the proteomic and lipid analysis, the resulting expression matrices were merged according to samples and evaluated by a weighted co-expression network analysis (WGCNA) as previously reported by Langfelder et al.^52^ An unsigned network was used with default parameters, except for β = 6, deepsplit = 4, Pearson correlation, minimal module size 10, pamStage = FALSE. Modules were correlated with clinical traits (timepoint, cycle, BOLD-100 dose) using a Pearson correlation and set into biological context by term enrichment analysis after mapping UNIPROT IDs to Gene Symbols.^56^

#### Description of statistical analysis

Permutation-based FDR was set to 0.05 for multi-parameter corrected significance testing (unpaired *t* test) of protein regulation. An additional fold-change boundary of Log_2_ fold-change >0.5 was set. For lipid species, statistical significance was calculated by an unpaired *t* test using the *limma* package and multiple testing correction was applied by the Benjamini-Hochberg procedure (FDR = 0.05).^50^ An additional fold-change boundary of Log_2_ fold-change >1 was set. ∗ = *p*-value <0.05 according to the multiple-testing corrected unpaired t-tests mentioned.

### Additional resources

Description: https://clinicaltrials.gov/study/NCT04421820.

## Supplemental information

Table S1. List of identified proteins and lipids

Table S2. List of features associated with each module in the co-expression calculation
